# Approaches to co-production of research in care homes: a scoping review

**DOI:** 10.1186/s40900-022-00408-z

**Published:** 2022-12-23

**Authors:** F. V. Hallam-Bowles, P. A. Logan, S. Timmons, K. R. Robinson

**Affiliations:** 1 Research and Innovation, Nottingham University Hospitals NHS Trust, Nottingham, UK; 2 Centre for Rehabilitation and Ageing Research, Injury, Inflammation and Recovery Sciences, Medical School, University of Nottingham, Nottingham, UK; 3 Nottingham CityCare Partnership, Nottingham, UK; 4 Centre for Health Innovation, Leadership and Learning, Nottingham University Business School, University of Nottingham, Nottingham, UK

**Keywords:** Barriers, Co-production, Co-creation, Co-design, Care homes, Facilitators, Participatory research, Social care, Stakeholder participation

## Abstract

**Background:**

Using the technique of co-production to develop research is considered good practice. Co-production involves the public, practitioners and academics working together as equals throughout a research project. Co-production may help develop alternative ways of delivering care for older adults that are acceptable to those who live and work in care homes. However, guidance about applying co-production approaches in this context is lacking. This scoping review aims to map co-production approaches used in care homes for older adults in previous research to support the inclusion of residents and care staff as equal collaborators in future studies.

**Methods:**

A scoping review was conducted using the Joanna Briggs Institute scoping review methodology. Seven electronic databases were searched for peer-reviewed primary studies using co-production approaches in care home settings for older adults. Studies were independently screened against eligibility criteria by two reviewers. Citation searching was completed. Data relating to study characteristics, co-production approaches used, including any barriers and facilitators, was charted by one reviewer and checked by another. Data was summarised using tables and diagrams with an accompanying narrative description. A collaborator group of care home and health service representatives were involved in the interpretation of the findings from their perspectives.

**Results:**

19 studies were selected for inclusion. A diverse range of approaches to co-production and engaging key stakeholders in care home settings were identified. 11 studies reported barriers and 13 reported facilitators affecting the co-production process. Barriers and facilitators to building relationships and achieving inclusive, equitable and reciprocal co-production were identified in alignment with the five NIHR principles. Practical considerations were also identified as potential barriers and facilitators.

**Conclusion:**

The components of co-production approaches, barriers and facilitators identified should inform the design of future research using co-production approaches in care homes. Future studies should be explicit in reporting what is meant by co-production, the methods used to support co-production, and steps taken to enact the principles of co-production. Sharing of key learning is required to support this field to develop. Evaluation of co-production approaches, including participants’ experiences of taking part in co-production processes, are areas for future research in care home settings.

**Supplementary Information:**

The online version contains supplementary material available at 10.1186/s40900-022-00408-z.

## Plain English Summary

Co-production involves people from different backgrounds working together as equals throughout a research project. Co-production may be a useful approach to help ensure that research in care homes focuses on approaches that are important and agreeable to older people and staff. A wide range of research and guidance about co-production has been published but there is limited guidance about how to do co-production in care homes. We carried out a review that involved pulling together previous research that used co-production in care homes for older adults. We looked at published research studies to learn about:

- Key components of the strategies used to achieve co-production,
- How care home residents and care home staff were involved,
- What helped or made co-production difficult to achieve.

A collaborator group including representatives from care homes and healthcare services were involved in this research. They helped decide what was most important about the results.

We found 19 published research articles that used co-production in care homes. The strategies used in the articles differed. There were also differences in how care home residents and staff were involved in co-production. Factors that helped people involved to work together in an inclusive and equal way were identified. At the same time, there were also many challenges.

These results should be used to design future research using co-production in care homes. Future studies should clearly report what is meant by co-production, the strategies used and key learning points. Evaluation of co-production and the experiences of people involved is needed.

**Supplementary Information:**

The online version contains supplementary material available at 10.1186/s40900-022-00408-z.

## Background

Many definitions of co-production exist and how the term is used often depends on a combination of factors, including the field in which it is applied, what is being produced, and the individuals and organisations involved [1, 2]. In this review, we consider co-production in health and social care research to be the involvement of service users, professionals and academics working together in equal partnership and sharing responsibility for generating knowledge and solutions to problems [3, 4]. Overarching guiding principles of co-production, such as power sharing, inclusivity, equality and reciprocity, have been developed; however, limited guidance based on empirical evidence is available regarding the practicalities of using co-production approaches in social care settings [3, 5, 6].

There is overlap between co-production and other terms, such as co-creation and co-design. These terms have originated from different fields and there are various lines of thought about whether they mean the same thing, or reflect different levels or types of involvement; however, it is generally accepted that all three terms involve the collaborative participation of multiple stakeholders in any or all stages of research [2, 7, 8]. Recent reviews have found co-methodologies have become increasingly popular over the last decade but there is variation in how such approaches are described and operationalised in health research [5, 9, 10]. Very few of the studies included in these reviews were conducted in social care settings such as care homes.

In the United Kingdom (UK), approximately 410,000 people live in care homes, many of whom are older adults with multiple health conditions and complex care needs, and demand is expected to increase due to the ageing population [11, 12]. Care homes also differ organisationally for many reasons, such as their ownership and commissioning arrangements, size, specialisms and culture [13]. Consequently, delivery of care in each unique care home setting requires a broad range of expertise and collaboration between stakeholders across numerous sectors in order to meet the individual needs and preferences of older care home residents [12].

By attending to these unique contextual factors, and harnessing the collective expertise and experiences of all stakeholders in care home settings, co-production research approaches may be more likely to be implemented and incorporated into routine practices in care homes [14]. However, achieving authentic co-production in alignment with its principles may be challenging and is likely to be influenced by many factors such as power relationships, social and cultural norms, and conflicting expectations and priorities. For instance, previous research has identified that patients accessing health services, the public and NHS staff perceived old age and poor health, both common characteristics of care home residents, as potential barriers to co-production [15]. This study did not appear to include care home staff or residents as participants. Barriers and facilitators to involving care home residents as Patient and Public Involvement (PPI) members in research have been identified, including social factors, organisational factors, skills and resources [16]. However, it is unclear whether the same factors would apply to co-production of research and whether there would be different factors to consider for involving other stakeholders, such as care home staff.

While co-production has been used in care homes in primary studies [17, 18], no reviews to date have focussed specifically on mapping the use of co-production in care home settings (to our knowledge). The aim of this scoping review is to map co-production approaches used in care homes for older adults to inform the design of future co-production research. The review sought to address the following questions:

- What co-production approaches have been used in care home settings for older adults?
- What are the key components of co-production approaches used in this context?
- What approaches were used to engage older residents and care home staff in the process?
- What barriers and facilitators to achieving co-production were reported?

## Methods

The review was undertaken following the Joanna Briggs Institute (JBI) scoping review methodology [19]. Protocol development and reporting was guided by the Preferred Reporting Items for Systematic reviews and Meta-Analyses extension for Scoping Reviews (PRISMA-ScR) checklist (Additional file 1) [20]. The protocol is published on the Open Science Framework [21].

### Search strategy

Eligibility criteria were developed using the Population, Concept, Context (PCC) framework and are outlined in Table 1 [19]. In this review, co-production was viewed as an umbrella term for describing stakeholders working together in equal partnership. We therefore included studies using co-production, co-creation or co-design as these terms are often used indiscriminately [7, 10].

**Table 1:** Eligibility criteria

Criteria	Inclusion	Exclusion
Population: care homes providing care for older adults	Care homes providing care for older adults defined as such by the authors (e.g. elders, older people, older adults)	Evidence focussing on care homes that provide care exclusively for people who are under the age of 65
Concept: co-production	Studies that explicitly state co-production, co-creation or co-design were used. The original authors’ assessment of the approach used was the basis for inclusion because we were interested in studies that had consciously set out to apply these approaches, and due to the variation in how these terms are conceptualised and operationalised in the literature, as described in the background to this review	Evidence focussing on involvement, engagement, or consultation of older care home residents or care staff without specific reference to co-production, co-creation or co-design
Concept: global care home settings	Research conducted in residential or nursing care home settings in any country	Research conducted in settings which do not provide permanent, 24-h personal care and support (for example home care, retirement communities, assisted-living, intermediate care or hospital-based settings)
Types of literature	Peer-reviewed primary studies using any quantitative or qualitative methods were included	Editorials, opinion pieces, protocols, systematic reviews and grey literature
Language	English language	Non-English languages
Date	Any year of publication	None

A comprehensive three-stage search strategy was conducted. Initial searches of MEDLINE and EMBASE were completed with advice from an information specialist at the University of Nottingham. Titles, abstracts and key terms from relevant texts identified through initial searching were analysed and used to create a tailored strategy for each information source based on variations of the following key concepts: older adults, co-production and care homes. An example search strategy is included in Additional file 2. A second search was completed in the following health and social care research databases on the 20th December 2021: AMED, ASSIA, CINAHL, EMBASE, MEDLINE, PsychInfo, Social Care Online. Thirdly, forwards and backwards citation searching of studies meeting the eligibility criteria was completed using Web of Science. Retrieved studies were imported into an EndNote X9 library.

### Study selection

Duplicates were removed using EndNote. Titles and abstracts were independently screened against the eligibility criteria by two reviewers (FHB, KR) using Rayyan [22]. Studies which used participatory research methods but did not explicitly state using co-production, co-creation or co-design in the abstract were included at this stage, as were studies which included terms with similar connotations to co-production (for instance, civil engagement, altruistic action) to minimise the risk of excluding relevant studies. Full texts were then screened independently by both reviewers and only included if use of co-production, co-creation or co-design was explicitly reported. Differences were resolved through discussion between the reviewers. Reasons for exclusion were recorded.

### Data charting

Data relating to co-production approaches used, involvement of key stakeholders, barriers and facilitators to achieving co-production reported in results or discussion sections, and key study characteristics were extracted from included studies. A data charting table (Additional file 3) was developed from a co-creation reporting checklist [26]. The checklist was used based on reviewers’ experiences of piloting the draft data charting table on five studies. Piloting was completed by two reviewers who found it difficult to systematically extract relevant data due to the heterogeneity of the approaches used across the studies. The co-creation checklist therefore helped to refine the structure and support a standardised approach to data charting [23]. For all included studies, one reviewer charted relevant data (FHB) and another checked the charted data for accuracy (KR). Discrepancies were resolved through discussion between the reviewers.

### Summarising and presenting findings

**Fig. 1:**
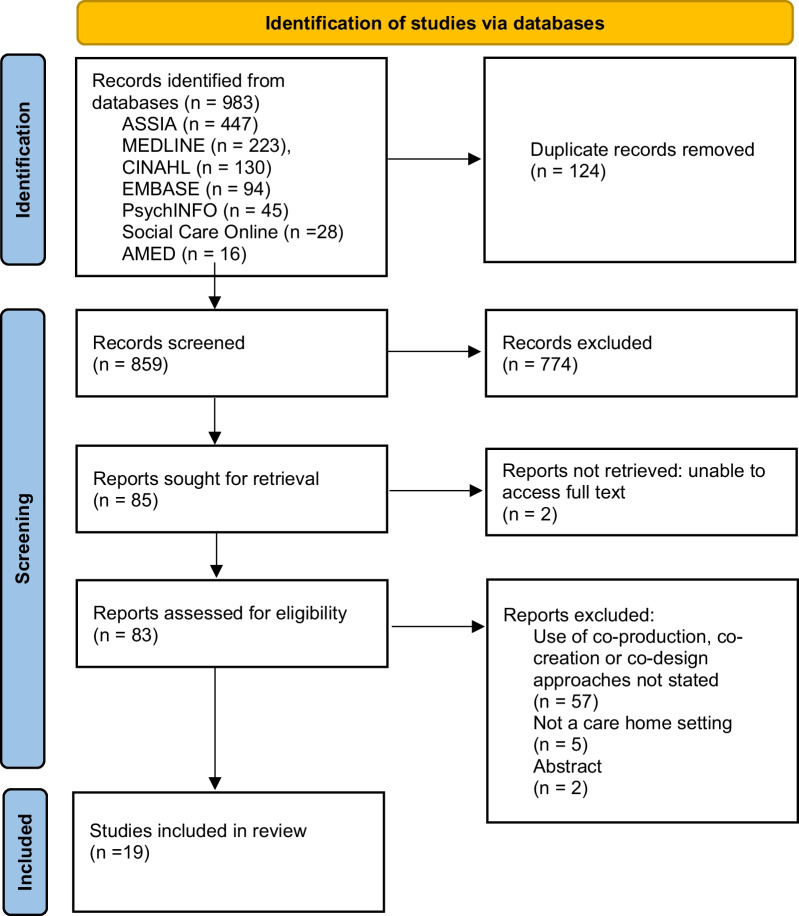
PRISMA flow diagram of the search strategy [24].

A PRISMA flow diagram was used to record the study selection process (Fig. 1) [24]. A narrative summary with accompanying tables and diagrams was developed to describe study characteristics, the co-production approaches used, their key components, and how care home staff and residents were involved. Reported barriers and facilitators to co-production were mapped against the NIHR principles of co-production [3] using a deductive, thematic analysis approach. The principles were originally developed by NIHR INVOLVE which is now part of the NIHR Centre for Engagement and Dissemination. Using an iterative approach, one reviewer (FHB) grouped barriers and facilitators from the included studies into themes based on similarity in meaning under the co-production principles. An “other” category was used for any factors that fell outside this framework. The themes and their placement under the co-production principles were then reviewed and revised by the second reviewer (KR), and finalised through discussion with the wider research team (PL, ST).

In keeping with the JBI scoping review methodology, studies were not critically appraised as the review was not intended to guide the selection of interventions for use in clinical practice [19, 25]. A collaborator group including care home, patient and public, and health care representatives were involved in decision-making about presentation of findings and their implications for future research. The group’s contribution to the review is reported using the GRIPP2 Short Form [26] in Additional file 4.

## Results

### Study selection

The process for identifying eligible studies is outlined in Fig. 1. Database searching yielded 983 records. After removal of 124 duplicates, titles and abstracts of 859 records were reviewed against the eligibility criteria. Of these, 85 were eligible for full text screening. Of the 83 that could be accessed, 19 were included in the review. Reasons for exclusion at the full text screening stage are provided in Fig. 1. No further studies were identified from forward and backwards citation searching.

### Characteristics of included studies

**Fig. 2:**
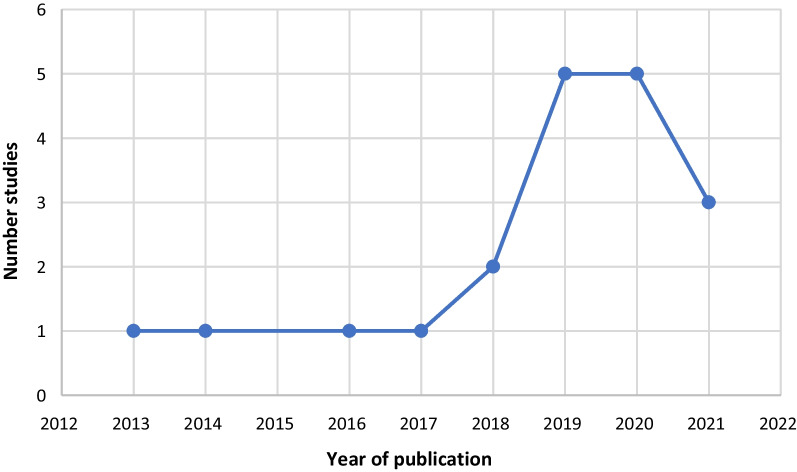
Line diagram of publications per year

Table 2 summarises the key characteristics of studies included in the review. The included studies were published between 2013 and 2021, with 68% (n = 13) published from 2018 onwards (Fig. 2). All studies were completed in high income countries and the lead author of most studies (n = 12) were based in the UK. 12 studies used qualitative research methods [17, 18, 27–36], six used mixed methods [37–42] and one was a descriptive piece about a collaborative partnership approach [43]. 15 studies reported using participatory research methodologies, with nine using participatory action research approaches [17, 18, 28, 29, 31, 35, 37, 38, 41] and four using appreciative inquiry [27, 30, 33, 39]. Collaborative enquiry [36], experienced-based co-design [42] and nominal group methods [42] were used once respectively.

**Table 2:** Characteristics of included studies

Reference	Lead author and year	Country of lead author	Aim	Methodologies and study design	Guidance used	Terminology used (co-production, co-creation or co-design)	Problem or topic addressed	Care home characteristics
[17]	Burns 2014	(UK)	To use participatory organisational research methods to actively involve residents, their relatives and the staff to explore mistreatment of older people in care homes	Qualitative- participatory organisational research including a literature review and comparative case studies Protocol panel development groups	Conceptualisations of voice- narrative research	Co-production	Mistreatment of older people in care homes	Care homes for people aged 65 years and over
[18]	Willis 2018	UK	To explore how co-production as a democratising approach to action-orientated research can emerge during the research and fieldwork process, and to reflect on the efficacy and ethical challenges of this approach for advancing a social inclusion agenda in care home settings for older people	Qualitative- community-based action research, evaluation Observation as part of an audit, informal meetings, formal advisory sessions, project meetings, semi-structured interviews, reflections	None reported	Co-production	Inclusion of older lesbian, gay, bisexual and transgender (LGBT)-identifying residents	6 residential care homes in a large city in England providing care for older people with a range of needs, some with complex physical disabilities and dementia
[27]	Curtis 2020	UK	To identify factors that enable nurses to implement digital health technology in nursing homes To co-design a nurse-led stepped process supporting the effective implementation of digital health technology innovations in nursing homes	Qualitative- appreciative inquiry Interviews, workshops	The 5 Ds cycle [44]	Co-design	Inconsistent implementation of digital health technology	5 care homes- varied in size, organisational structure and previous digital health technology use
[28]	de Boer 2020	Netherlands	To report on the co-creation of an alternative nursing home model	Qualitative- participatory research, case study Project meetings	Cites the co-creation process as described by Bergdahl et al. [45] and a systematic review [46]	Co-creation	Development of a new nursing home model and dementia care environment	Large care provider in the south of the Netherlands who were setting up a new care home in a rural village location Part of the Living Lab in Ageing and Long-term Care (collaboration between research, education and care organizations in the Netherlands)
[29]	Demecs 2019	Australia	To explore if and how creative occupation, a participatory art project, might benefit older people living in residential aged care	Qualitative- participatory research, longitudinal case study Weaving participation, workshops, interviews	Interpretative phenomenological analysis – grounded in phenomenology	Co-creation and co-design	Limited opportunities to engage in meaningful craft occupation	1 not-for-profit residential aged care facility in Queensland. 200 rooms spread across separate buildings with a pool, birdhouse, coffee shop and hair salon
[30]	Dewar 2017	UK	To explore the relevance of a framework to the care home setting and the development of an educational intervention, based on the framework, to enhance development of human interaction	Qualitative- appreciative inquiry Meetings, observations, discussions, group interviews, exit interviews, photo-elicitation	Appreciative inquiry process, the Caring Conversations Framework [47]	Co-creation	Development of an educational intervention based on the framework	1 care home in Scotland with 4 units, registered for 72 residents and employer of 100 staff
[31]	Dugstad 2019	Norway	To identify facilitators and barriers, and to explore co-creation practices as an innovation strategy during four years of implementation of a digital monitoring technology in long-term residential care for persons with dementia who were night wanderers	Qualitative- transformational action research, longitudinal case study Workshops, individual interviews, focus groups	Triple-helix model	Co-creation	Resistance to implementing digital monitoring technology for people with dementia who were night wanderers	8 nursing homes with dementia care wards
[32]	Fowler-Davis 2021	UK	To understand the impact of the universal enhanced support offer to care homes using co- production methods, appreciative inquiry and analysis	Qualitative- appreciative inquiry, evaluation Interviews, focus groups, workshops	Appreciative inquiry and 4 D cycle (Discover, Dream, Design, Destiny) [48]	Co-design (although co-production mentioned in the aim of the study)	Support requirements of care homes due to the challenges of the Covid-19 pandemic	Care homes in the North East and Yorkshire region
[33]	Hafford-Letchfield 2020	UK	To explore the discourse of ‘giving up’ from the perspective and understanding of care home staff To identify factors they perceived to either prevent or contribute to such situations emerging To highlight best practice responses	Qualitative- descriptive and exploratory study Workshop, focus group	None reported	Co-design (although co-production mentioned in the abstract)	Limited understanding and literature exploring the concept of ‘giving up’ among older people in care homes	4 care homes in the South-East region of England 2 private sector (1 recently acquired nursing home status) and 2 charity sector homes All had a ‘good’ CQC rating and cared for adults over 65 years of age
[34]	Jamin 2018	Netherlands	To describe the challenging design process, in co-creation with the artist and all stakeholders, of the interface of VENSTER, an interactive artwork for nursing home residents, and share the used methods during and lessons learned from this design process	Qualitative Workshops, activity card sort, useability testing (Think aloud method and Wizard of Oz technique)	None reported	Co-creation	Involving residents in the design of interactive artwork	None reported
[35]	Prentice 2021	New Zealand	To explore and act on factors that encourage caregivers to be engaged and motivated in their work with older adults in aged residential care	Qualitative- participatory action research Advisory group meetings, interviews, feedback meeting	Lewin’s action research model- plan, act, observe, reflect [49]	Co-design	Workplace engagement of caregivers	1 40-bed rural aged residential care facility providing rest home- and hospital-level care
[36]	Watson 2020	UK	To co-create curricular content on care home nursing with student nurses	Qualitative- collaborative enquiry Focus groups, interviews	A theoretical framework of co-creation through collaborative enquiry [50]	Co-creation	Embedding care home nursing in the student nursing curriculum	Not applicable
[37]	Gine-Garriga 2019	UK and Spain	To integrate service-learning methodology into University degrees by offering students individual service opportunities with residential care homes To co-create the best suited intervention to reduce the sedentary behaviour of residents throughout the day, with researchers, end-users, care staff, family members and policymakers	Mixed methods- participatory action research, service learning methodology Workshops, collection of activity data	None reported	Co-creation	Sedentary behaviour of care home residents	4 care homes- 2 from Glasgow, 2 from Barcelona
[38]	Griffiths 2021	UK	To work with care home staff to create a learning culture to address how to promote mouth care for residents	Mixed methods- participatory research, service evaluation and development Academic partnership approach, meetings, survey	None reported	Co-production	Sub-optimal mouth care for residents	Care homes in the North of England who were part of a partnership (NICHE-Leeds) between academia and care organisations
[39]	Manthorpe 2013	UK	To report and reflect on a recently completed five-year programme of research on dementia care and practice in England	Mixed methods- appreciative inquiry, case study Interviews and focus groups mentioned in studies that reported inclusion of care homes	Appreciative inquiry	Co-design	Support and resources for people with dementia and their carers (End of life care and implementing the MentalCcapacity Act specifically in care homes)	Care homes covered by the Central and North West London NHS Trust and in its neighbouring areas
[40]	Treadaway 2016	UK	To present design research investigating the development of sensory textiles with embedded electronics to support the wellbeing of people with late stage dementia in residential care	Mixed methods- grounded practical theory methodologies Case study interviews, workshops, unstructured interviews, observations	Positive design framework	Co-design	Sensory textiles for residents with late stage dementia	3 residential care homes owned by Gwalia (major provider of social care in Wales) with specialist dementia units
[41]	Patel 2019	UK	To pilot co-production, delivery and evaluation of an oral care training programme with care home staff	Mixed methods- action research In-depth interviews, group discussions, informal discussions, pre- and post- questionnaires	Social Care Institute for Excellence principles of co-production- culture, structure, practice and review [51]	Co-production	Lack of oral care training for care home staff	3 care homes in Central London from NHS, local authority and private sectors
[42]	Pownall 2019	UK	To explore the knowledge needs of the workforce and develop a resource to meet this need, in order for them to be able to identify the presence of dysphagia in their residents and to understand how to manage dysphagia holistically	Mixed methods- experienced based co-design consensus methodology, usability testing methodology, agile methodology adopted for the software development Focus groups, semi-structured interviews, nominal group method, user-testing, questionnaire, digital analytics	Experienced-based co-design	Co-design (although co-production mentioned in the abstract)	Management of dysphagia	4 nursing homes in a city in the north of England including corporate and small-to-medium-sized enterprises with residents with dysphagia, registered for nursing and/or residential care
[43]	Luijkx 2020	Netherlands	To describe how co-creation, in the sense of close, intensive, and equivalent collaboration between science, care practice, and education, is a key factor in the success of improving long-term care for older adults	Not reported (descriptive piece) Academic partnership approach, various designs depending on the projects	None reported	Co-creation	Person- centred care	South and South West of Netherlands and linked to an academic and care partnership

### Approaches to co-production and their components

#### Descriptions of approaches

A mixture of co-production, co-creation and co-design approaches were used. Table 3 outlines how these terms were defined across the included studies. All studies (n = 4) using co-production approaches provided a definition [17, 18, 38, 41], as did most of the studies (n = 6) using co-creation [28, 29, 31, 36, 37, 43]. However, only two out of eight studies using co-design approaches included a definition [29, 42] and three studies used the term co-design interchangeably with co-production [32, 33, 42]. Co-design was described as an approach centred on valuing lived experiences at any or all stages of research in order to understand needs and develop a useful product. Co-creation was defined as a collaborative approach involving stakeholders or end-users in the shared creation of outputs that were mutually valuable. In comparison to co-design, co-creation was used to develop a wider range of outputs, ranging from knowledge to outcomes. While definitions of co-production also emphasised collaborative working between stakeholders, these definitions incorporated the concept of equality. Equality was considered in relation to partnership working, respecting knowledge and disrupting power asymmetries.

**Table 3:** Terms and definitions used to describe approaches

Term used	Number of articles providing a definition	Definitions used
Co-creation	6 out of 8	• “The joint creation of vital goals for patients through the process of sharing knowledge and values” (p.3) [28] • Examples of co-creation given to explain their approach [29] • “An interaction where actors jointly produce a mutually valued outcome based on assessments of the risks and benefits of proposed courses of action and decisions based on dialogue, access to information and transparency” (p.3) [41] • “shifts the design process from the traditional “top-down” health model to an inductive paradigm of shared leadership allowing end-users to take control over the content of the activities, and be involved in their health management and decision-making relevant to their own health” (p.2) [37] • “Close, intensive and equivalent collaboration between science, care practice and education in the development of innovative, evidence-based knowledge” (p.2) [43] • “In co-creation through collaborative enquiry, student learners can become meaningful contributors to the planning and approval processes of programme and course content in developing a nursing curriculum responsive to population needs” (p.1) [36]
Co-design	2 out of 8	• Examples of co-design given to explain their approach [29] • “Co-design methods have been variously defined, but, in this case, the ambition was to enable a detailed understanding of functionality of the learning needs of care home staff and modelling of a physical system to convert this into product ‘architecture’. Using an experience-based co-design process, the participants can be involved in all stages or simply offer an interview, but recognise their engagement as valuing the lived experience of receiving or delivering care” (p.3) [41]
Co-production	4 out of 4	• “the results of mutual engagement are commonly referred to as having been co-produced” (p.133) [17] • “working together and recognising different forms of knowledge” (p.3) [38] • “The Social Care Institute for Excellence [51] defines co-production as ‘people who use services and carers working with professionals in equal partnerships towards shared goals’” (p.164) [41] • ““unsettling traditional relations between expert and public knowledge” (p. 145) [52] and disrupting the more conventional power asymmetry between researcher and those researched. As a methodology, this approach to research provides a democratising platform for the inclusion of multiple parties involved in the production of knowledge (university researchers, user/participant groups, community organizations, for example)” (p.3) [18]

### Focus and topics

Co-production approaches were used to explore a diverse range of problems or topics across multiple fields such as the arts, design, technology and health. The focus of the co-production approaches broadly aligned into three categories (Table 4). Four studies used co-production or co-design to increase understanding or explore a given topic area [17, 18, 32, 33]. Nine studies used co-creation or co-design to develop new ways to deliver care [27–29, 31, 34, 37, 39, 40, 43]. These studies specifically focused on dementia care, implementing digital technology, art-based approaches, and individualised approaches. Six studies used co-production, co-creation or co-design to support care home workforce development [30, 35, 36, 38, 41, 42], with five co-producing outputs aimed at developing capabilities and one addressing motivation at work.

**Table 4:** Focus of co-production and topics addressed

Focus of the co-production	Problem/topic addressed
Increasing understanding/knowledge production (n = 4)	Mistreatment of older people in care homes [17] ‘Giving up’ among older people in care homes [33] Inclusion of older LGBT-identifying residents [18] Support requirements of care homes during the Covid-19 pandemic [32]
Developing new approaches to delivering care (n = 9)	**Dementia care:** Development of a new nursing home model and dementia care environment [28] Sensory textiles for residents with late stage dementia [40] Support and resources for people with dementia and their carers [39] Design of interactive artwork [34] **Implementing digital technology:** Inconsistent implementation of digital health technology [27] Resistance to implementing digital monitoring technology for people with dementia [31] **Arts-based approaches:** Limited opportunities to engage in meaningful craft occupation [29] Design of interactive artwork [34] Individualised approaches: Sedentary behaviour of care home residents [37] Person- centred care [43]
Developing the care home workforce (n = 6)	**Capabilities:** Educational intervention based on the Caring Conversations framework [30] Sub-optimal mouth care for residents [38] Lack of oral care training for care home staff [41] Management of dysphagia [42] Embedding care home nursing in the student curriculum [36] **Motivation:** Workplace engagement of caregivers [35]

### Stakeholder involvement

**Fig. 3:**
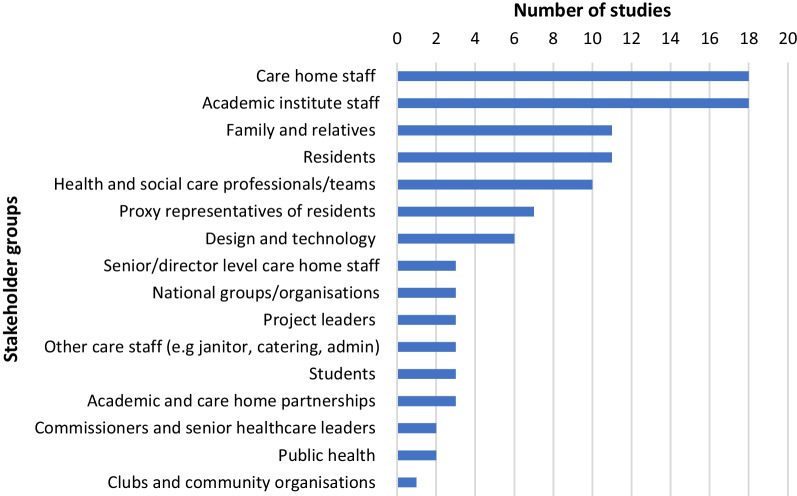
Bar chart of stakeholder group involvement

Stakeholder involvement in the co-production process varied across the studies (Fig. 3). Care home staff and academic institute staff were most often involved (n = 18), followed by residents (n = 11), family and relatives (n = 11) and health and social care professionals or teams (n = 10) respectively. Some studies (n = 7) included proxy representatives for care home residents, such as older people. Other stakeholder groups were linked to the problem or topic area to be addressed, such as design and technology staff.

### Stages and levels of stakeholder participation

**Fig. 4:**
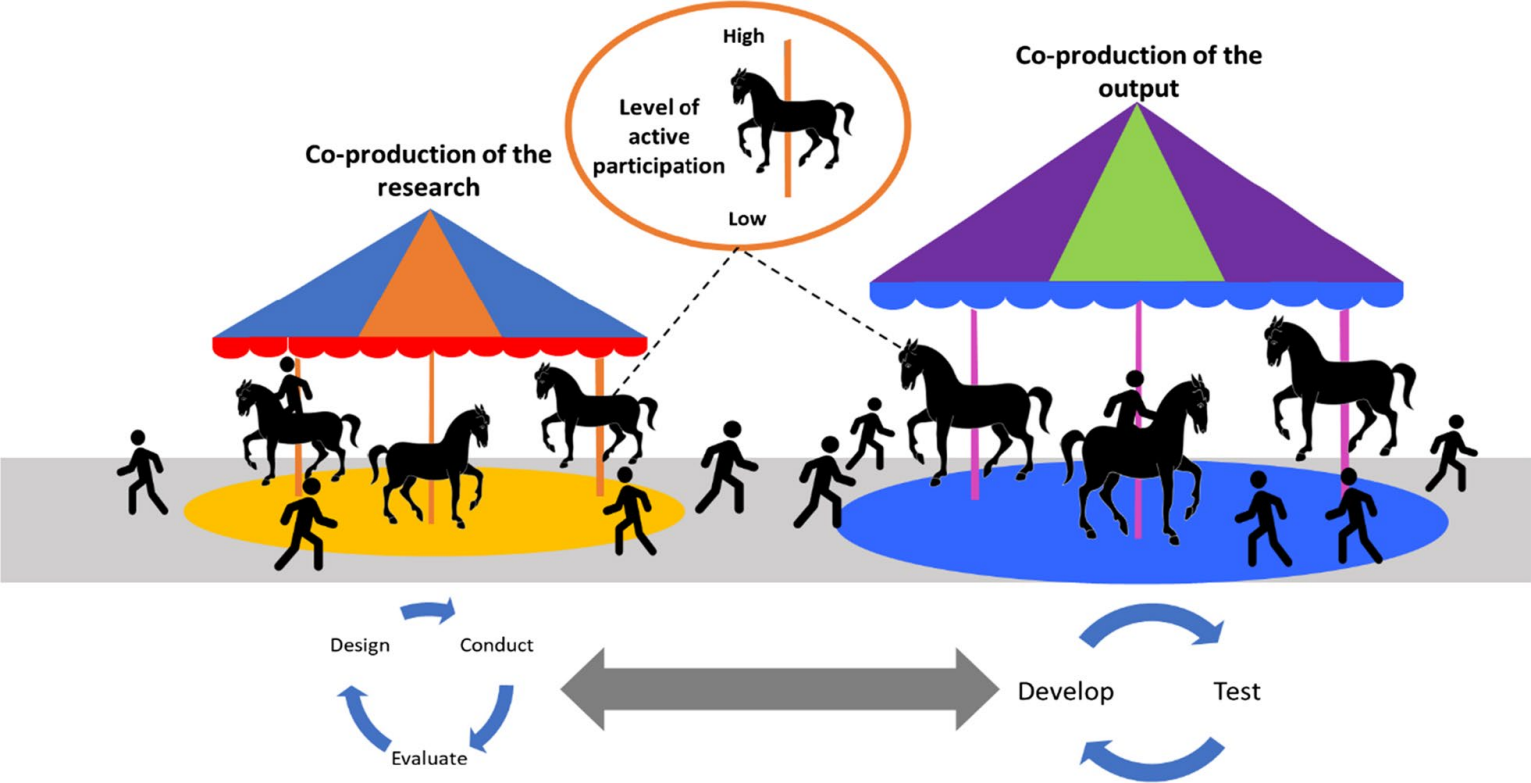
Carousels of co-production

Stakeholders participated in co-production at various stages as represented by the image below of two carousels connected by a path (Fig. 4). The carousels depict how stakeholders participated in the cyclical process of co-producing the research project, or of co-producing the intended output, or in elements of both processes. Outputs of co-production in the included studies ranged from conceptual knowledge or models to tangible products.

**Fig. 5:**
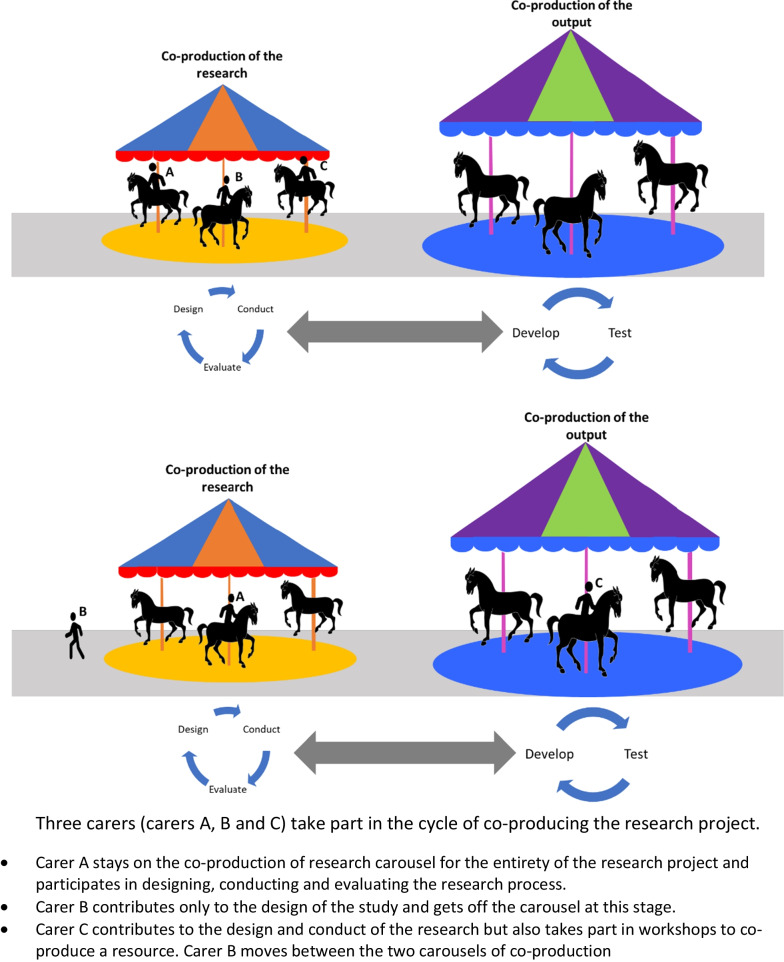
Illustrative example of stakeholder participation in co-production processes

There was variation in the points at which stakeholders became involved or ceased involvement in each co-production cycle, shown by the stakeholders on the diagram getting on and off the co-production carousals at different points. An illustrative example is provided in Fig. 5

For the research process, stakeholders were most often reported to participate in conducting the research (n = 10) [17, 18, 28, 30, 32, 35, 37–39, 43] and less commonly reported to be involved exclusively in the design (n = 3) [17, 39, 41] or evaluation (n = 5) [18, 29, 35, 37, 39] stages of the co-production research process. Stakeholders were commonly involved in the co-production of the output, mostly in the development stage (n = 15) [17, 18, 27–31, 33, 34, 36–38, 40–42]. Stakeholders tested early iterations or prototypes in some studies (n = 8) [30–32, 34, 35, 40–42].

Involvement of stakeholders across the studies differed. There was no pattern in the stages that stakeholders were involved in based on the use of the terms co-production, co-creation or co-design. Sometimes reporting of involvement seemed to be linked to the article’s aim and the context and timelines of research programmes. For instance, Manthorpe at el [39]. aimed to reflect on the completion of a large five year research programme and report involvement in all stages of the co-production research process. Other studies [27, 36], appeared to be smaller projects with an aim to develop a specific output and reported involvement of stakeholders exclusively in co-production of the output through participation in interviews and workshops. Stages of involvement in the included studies were sometimes difficult to decipher based on the information reported in the original studies. For instance, Fowler-Davis et al. [32] reported a co-produced evaluation of an approach to support care homes during the Covid-19 pandemic. NHS and care home staff were then involved in sharing their experiences of the approach. While the authors state the approach itself was collaborative, it is unclear whether it was also co-produced. Thus, some of the variation seen may be due to differences in reporting.

The level of active stakeholder participation in the studies, on a sliding scale of high to low, is depicted by the horses moving up and down on the carousels. The degree to which stakeholders were supported to participate as equal partners in alignment with key principles of co-production was rarely reported. Where information was provided, the level of participation appeared to vary. For example, in some studies stakeholders attended co-creation workshops to contribute their ideas or perspectives but the topics and format of the discussion were set by the research team [29, 34]. In other studies, stakeholders were actively involved in deciding the focus of discussions, as well as contributing their ideas and perspectives, suggesting a higher degree of stakeholder involvement [18, 28].

### Approaches to engaging care home staff and residents

#### Co-production of the research

Care home staff in various roles, residents and groups representing residents were involved in the co-production of the research using numerous approaches. Residents, older people and care home staff were part of research advisory groups involved in designing research projects or programmes [17, 28]. Most commonly (n = 9), staff and groups who represented residents were involved in conducting the research as a member of a core research group [18, 28, 30, 32, 35], as a co-researcher [17, 36, 39, 43], or through an established academic-care collaboration [38, 43]. Five studies involved residents, groups who represent residents and staff in evaluating the co-production process. This was done by conducting semi-structured interviews about their experiences of the process [18, 29, 35], incorporating stakeholder reflections into the evaluation [17, 39], or through involvement in data analysis [37, 39].

#### Co-production of the output

Care home staff, residents, and representatives of residents were more often involved in the process of co-producing the output in comparison to co-producing the research. A wide variety of methods were used across 16 studies to engage staff and residents in developing the output [17, 18, 27–31, 33–38, 40–42]. The most common approach was to hold a series of workshops or meetings [18, 27, 29, 33, 34, 37]. Similarly, there was heterogeneity in approaches to engage staff in testing the output across seven studies [30–32, 35, 40–42], with the most common approach being one-to-one interviews. In contrast, only two studies involved residents, and specifically residents with cognitive impairments, in testing the outputs and both used user-testing methods [34, 40].

### Barriers and facilitators to achieving co-production

Barriers to in 11 studies [17, 18, 29–31, 33, 34, 37, 39, 41, 42] and facilitators of co-production were reported in 13 studies [17, 18, 29–31, 34, 35, 37–40, 42, 43]. As few studies formally evaluated the co-production process, these were mostly reported in the discussion sections of included studies.

Barriers and facilitators to achieving each NIHR co-production principle [3] are provided in Table 5 with specific examples explained below. Barriers and facilitators are presented according to the principle that the research team felt they most strongly resonated with; however, it should be noted that they often related to more than one principle as the principles are interconnected. Barriers and facilitators are presented in the same manner as they were portrayed in the original articles.

**Table 5:** Barriers and facilitators to achieving the NIHR principles of co-production [3]

NIHR principle	Barriers	Facilitators
Sharing power	**Burden of supporting resident involvement on care staff** [39, 41] Recruiting and gathering perspectives of residents during the research placed additional pressure on care home staff. This limited resident involvement **Gatekeeping** [39, 42] Care home staff and health professionals supported recruitment of residents into some research studies and likely influenced who had access to opportunities to be involved **Ethical procedures** [17] Ethical processes, such as formal signed consent, may have reinforced power imbalances and restricted or deterred resident participation in co-production **Delineating roles in the research process** [17, 39] Involving stakeholders in different roles, such as panel members, peer researchers and research participants, created hierarchies among those involved in co-production and sometimes led to ambiguity about ownership of the research	**Creating opportunities to challenge dominant views** [17, 18, 39] Opportunities and positions were created which supported marginalised groups, such as residents, to challenge dominant views and helped reduce hierarchies. This was achieved through the creation of expert roles, safe spaces for non-judgemental dialogue and through the ethos of co-design in general **Reflexivity of project leads and researchers** [18] Project leads and research teams who exhibited reflexivity were responsive to the perspective of stakeholders and able to relinquish control of the project despite the uncertainty that this created
Including all perspectives	**Not enough involvement of key stakeholders** [18, 31, 41, 42] Key stakeholders were sometimes not involved early enough, in large enough numbers, or at all **Pressures on care home staff and healthcare professionals** [18, 37–39] Seeking perspectives of busy care home staff and healthcare professionals was challenging. This was due to the daily pressures of their roles and the complex nature of the contexts they work in, where staff, priorities and organisational structures change frequently and unpredictably **Care home resident characteristics** [37, 39] Characteristics specific to care home resident populations were identified as barriers to recruitment and opportunities to share perspectives. This included having a diagnosis of dementia, fatigue and reduced concentration **Limited depth of discussion** [31, 37] The depth of discussions and insight into perspectives was sometimes limited due to lack of time available or inexperience of student facilitators **Difficulties with stretching perspectives** [18, 34] It could be challenging for stakeholders to consider other perspectives that were different to their own, or to see the bigger picture	**Stimulating experiences** [29, 40] Stimulating experiences supported involvement from a wide range of stakeholders. Bringing a range of stakeholders together in a creative, unfamiliar environment facilitated an enjoyable experience. Connecting objects and handling materials helped to stimulate participation from people with dementia **Care home staff’s willingness to participate** [18, 38] Care home staff were willing to contribute their views and engage in conversations about sensitive topics. Buy-in from senior managers supported staff to be involved in all stages of the research **Flexible approach** [18, 34, 40] Flexible and iterative approaches facilitated deeper understanding of perspectives, incorporation of different methods and expertise, and the development of outputs that met the needs and preferences of the end-users
Respecting and valuing knowledge	**Lack of self-confidence** [18, 30, 37] Care home staff, residents and stakeholders representing residents described a lack of confidence in their ability to contribute to the co-production process **Balancing different forms of knowledge** [28, 37] Sometimes different knowledge led to “trade-offs”, such as balancing the researcher’s knowledge of the evidence-base with practical considerations of care homes, and developing strategies based on resident preferences which are also feasible to deliver	**Involvement across design stages** [42] Involving care home staff throughout all the design stages helped staff feel that their knowledge was valued and see the contribution that they made to the process **Recognising and utilising different forms of expertise** [17, 18, 29, 31, 40, 42] Projects that recognised different forms of knowledge and expertise used this to shape the research and develop resources that were useful and accessible in care home contexts. This included utilising experiential, subject-specific, organisational and political knowledge
Reciprocity	**Potential harms of participation** [17, 18] Participating in co-production could cause memories of painful experiences to resurface and evoke anxiety when challenging the views of other stakeholders. Some were cautious about voicing their perspective due to potential negative consequences of doing so	**Providing support** [18] In one study [18], advisors were provided with emotional and practical support by the project leader and by working in pairs with another advisor **Providing learning opportunities** [18, 28, 35, 40] Stakeholder learning was supported as part of the process, through structured activities, trying out new ideas in their care home, or learning from the expertise and experiences of other stakeholders **Clarifying expectations** [38] Being clear about expectations, including short- and long-term outcomes, was important to avoid issues later down the line
Building and maintaining relationships	**Relationships with management** [18, 31, 41] Making initial contact and maintaining relationships with care home managers could be difficult. Sometimes management or ownership of the care home changed during the study which led to changes in organisational priorities **Optimising links with wider stakeholders** [37, 39] Links with wider stakeholders and partners, such as NHS PPI structures and community organisations, were not always well-defined and there may have been missed opportunities for collaboration **Differences between stakeholders** [18, 31, 35, 37, 39] Stakeholders often had different beliefs, cultures and organisational norms. This created difficulties with communication, sharing opposing views and developing understanding between stakeholder groups **Practical challenges** [18, 31, 39] Practical aspects of the projects, including short project timescales, de-prioritisation of reflection and difficulties planning engagement activities, presented challenges to building report and maintaining stakeholder motivation throughout the project	**Building and utilising existing partnerships** [38, 39] Investing in building relationships and utilising existing partnerships helped to engage stakeholders in conversation and in the research process **Regular meetings and dialogue** [18, 31, 39] Having regular meetings was important for facilitating dialogue and building connection between stakeholders. Regular meetings were also helpful for research teams working across large research programmes **Establishing ways of working** [31, 39, 40] Recognising different ways of working and developing acceptable approaches which accommodated individual preferences facilitated collaboration between stakeholders **Project leadership** [31, 35, 37, 39] Leaders, including named project leaders, programme research managers, champions in care homes, and students, acted as knowledge brokers who helped to facilitate dialogue with stakeholders and resolve problems. Projects led by researchers with past experience of working in the care sector enabled rapport to be built with care home staff **Connection through creative approaches** [29, 30] Creative approaches enabled relationships between residents and staff to shift and build through the slow pace of creative activities. In one study, continued engagement was facilitated through displaying the finished tapestry in the care home^29^ **Sustaining relationships through participatory approaches** [39] The participatory approach itself, such as appreciative enquiry or co-design, was reported to have a positive impact on future interactions between stakeholders and sustaining relationships
Other	**Feasibility of scaling co-production** [41] It may not be feasible to co-produce interventions or deliver co-produced outputs on a larger scale	**Logistical arrangements** [18, 31, 39, 43] Resources, such as time and funding, were required to conduct co-production activities. Maintaining autonomy of projects within programmes of research and immersion of the researcher in the process enabled projects to be responsive

Bold text indicates the themes relating to barriers and facilitators to achieving the principles of co-production

Some barriers and facilitators are closely related. For instance, balancing different forms of knowledge was a reported barrier, whereas recognising and utilising different forms of knowledge was a facilitator.

#### Sharing power

Sharing power relates to shared ownership of research [3].

Four barriers to achieving this principle were identified from four studies: *the burden of supporting resident involvement on care staff* [39, 41]*, gatekeeping* [39, 42]*, ethical procedures* [17]*, delineating roles in the research process* [17, 39]. For example, one study [17] discussed how ethical procedures, such as formal signed consent, may have reinforced power imbalances by categorising older people as vulnerable or dependent, and restrictions on how residents could participate deterred some from taking part.

However, two facilitators of power sharing were reported in three studies*: creating opportunities to challenge dominant views* [17, 18, 39]*, reflexivity of project leads and researchers* [18]*.* For instance, expert roles helped to challenge dominant views by elevating the social status of residents, enabling authority and opportunities to be involved in discussions [17]. Provision of opportunities for critical reflection and discussion was reported as beneficial in a study exploring LGBT inclusion [18].

#### Including all perspectives and skills

This principle relates to ensuring that research teams involve diverse experiences, expertise and skills [3].

Five barriers relating to inclusivity were noted across eight studies: *not enough involvement of key stakeholders* [18, 31, 41, 42]*, pressures on care home and healthcare professionals* [18, 37–39]*, care home resident characteristics* [37, 39]*, limited depth of discussion* [31, 37]*, difficulties with stretching perspectives* [18, 34]*.* Focusing on characteristics of residents as an example, recruitment of people with dementia was described as challenging due to reduced cognition and stigma associated with this condition [37, 39]. Fatigue and low concentration levels led to less time to cover planned topic areas in workshops [37].

In contrast, three facilitators to inclusive co-production were identified from five studies: *stimulating experiences* [29, 40]*, care home staff’s willingness to participate* [18, 38]*, flexible approaches* [18, 34, 40]*.* Stimulating experience was specific to studies that involved stakeholders from the fields of arts and design. For instance, Treadaway et al. [40] held an event in an art studio with multiple stakeholders, including older people, family members, carers, health professionals, and designers, and provided sensory stimulation through handling materials. This was reported to facilitate creativity and fun by attendees.

#### Respecting and valuing knowledge

This principle involves viewing and appreciating all types of knowledge as equal [3].

Two challenges to this principle were reported across five studies: *lack of self-confidence* [18, 30, 37]*, balancing different forms of knowledge* [28, 37]*.* Lack of self-confidence affected a range of stakeholders. One study described residents’ “feelings of being useless” (p.11) [37]. Similarly, advisors in another study described feeling “out of their depth” (p.6) [18]. Care home staff lacked confidence in their influencing skills [30].

*Involvement across design stages* [42]* and recognizing and utilising different forms of knowledge* were facilitators to respecting knowledge across six studies [17, 18, 31, 39, 40, 42]. The latter involved valuing the experiential, subject-specific, organisational and political knowledge held by different stakeholders as assets.

#### Reciprocity

Reciprocity refers to benefitting from participation in co-production [3].

*Potential harms of participation* were noted in studies exploring difficult experiences and discrimination. For example, advisors who identified as LGBT in Willis et al.’s [18]. study described re-living painful experiences and feeling anxious when challenging beliefs of other participants. Older adults were worried there may be negative consequences to sharing their experiences regarding mistreatment of residents [17].

However, two facilitators to reciprocity were noted in five studies: *providing support* [18]*, providing learning opportunities* [18, 29, 35, 40]*, clarifying expectations* [38]*.* Using Willis et al. [18] as an example, advocates were provided emotional and practical support through regular contact with the project leader and received peer support through working in pairs to mitigate against potential harms. Learning opportunities were provided through structured training to provide background to the project and build connection between advisors.

#### Building and maintaining relationships

Relational working is a core component of co-production [3].

Four barriers to this concept were noted in six studies and related to: *relationships with management* [18, 31, 41]*, optimising links with stakeholders* [37, 39]*, differences between stakeholders* [18, 31, 35, 37, 39]*, practical challenges* [18, 31, 39]*.* Differences between stakeholders was most often mentioned. Several studies mentioned a lack of understanding between stakeholders due to differences in language, theories, moral beliefs, and professional and organisational cultures [18, 31, 35, 39].

More often, six facilitators regarding relationships were noted across nine studies*: building and utilising existing partnerships* [38, 39]*, regular meetings and dialogue* [18, 31, 39]*, establishing ways of working* [31, 39, 40]*, project leadership* [31, 35, 37, 39]*, connection through creative approaches* [29, 30]*, sustaining relationships through participatory approaches* [39]*.* To support relational working between different stakeholders, approaches such as developing common language [31], processes and outcomes [39] were used. However, in Treadaway et al.’s [40] project, establishing ways of working involved recognising that one stakeholder group (technologists) preferred a different way of working to other stakeholders.

#### Other practical considerations

Practical considerations that did not fit within the principles of co-production were captured by some studies. Patel et al. [41] highlighted that *feasibility of co-production on a wider scale* may be challenging and reflected on concerns regarding the practicalities of involving a larger number of care homes in the co-production process and providing their co-produced training on a larger scale. In contrast, four studies identified *logistical arrangements* that may facilitate co-production. Access to time and resources was most commonly mentioned; this related to optimising the success of workshops [31], opportunities for reflexivity [18] and expanding research programmes [43].

## Discussion

This scoping review identified that research using co-production approaches in care homes is limited. Most studies employed qualitative methods and participatory methodologies. Research was conducted across a variety of fields, involved multiple stakeholders, and addressed a wide range of topics of relevance to care home residents and staff. A range of approaches, such as workshops, interviews and focus groups, were used to engage care home residents and staff in co-production; however, the co-production process or the co-produced output was rarely evaluated. The review has identified setting-specific insights into using co-production in care homes, such as identification of key stakeholders, approaches for involving them in research, and identification of potential barriers and facilitators when applying principles of co-production in this context. These findings could be used to inform the design of future studies and to actively involve residents and care home staff in this process.

### Wider context

In line with other reviews exploring co-production in health and social care research [5, 53], we identified growth in publications using co-production in care home settings over the last decade. Whereas co-production research conducted in the health field began to appear as early as the 1990s, [5] the earliest study identified in this review was published in 2013. This suggests that there is a lag in the use of co-production approaches between health and care home settings therefore continual investigation of co-production in care home contexts is required. The 2021 UK Government social care policy white paper ‘People at the Heart of Care’ [54] directly references co-production on multiple occasions therefore it is likely that the co-production research in care home settings will continue to accelerate in the UK in the coming years.

Heterogeneity in reporting of definitions and the underpinning co-production principles was also a common feature of the studies included in the review. This concurs with a scoping review of definitions of co-production and co-design in health and social care research which found a third of included articles provided no definition or explanation [53]. This review also identified an increase in the number of new definitions and an increase in the number of publications using the terms co‐production or co‐design while not involving patients or the public over the last decade [53]. There is much debate about the need for standardised definitions. Concerns have been raised regarding broad definitions which may have the potential to be misappropriated [55]. On the other hand, it has been suggested that a looser definition of co-production allows more flexibility to adapt approaches depending on the context, and holding research against rigid principles may be more harmful by inadvertently discouraging participation through setting unattainable standards [3, 9, 56]. Based on our experiences of completing this review, and in alignment with the conclusions of reviews in other settings [9, 10, 53], we suggest clearer reporting about how authors define and operationalise co-production in care home settings would be a pragmatic compromise which allows for flexibility while also aiding the reader’s assessment of authenticity. Reporting frameworks such as the one that guided the data extraction process in this review may be helpful [23]; however, based on our experiences of using the framework to support data charting, further refinement may be needed if the framework is to encapsulate all approaches that fall under the co-production umbrella. Some aspects of the framework could be streamlined to avoid repetition while others require further explanation. Reporting of co-production research is therefore an area for further development.

Evaluation of the co-production process, how well stakeholders were engaged in the process, and the output of co-production was lacking in the included studies. This is similar to findings of other reviews and ethnographic research which focussed on co-production in health settings rather than social care settings [9, 10, 57, 58]. Further exploration of how to evaluate co-production approaches and outputs of co-production is required. Reporting and reflection on the process of co-production may support others to learn from past research. The collaborator group involved in this review were also surprised at the lack of reporting about the emotional component of co-production and how it feels to be a co-researcher or stakeholder working in the space of co-production. Exploration of experiences’ of PPI in research and clinical commissioning group (CCG) activities identified mixed feelings among PPI representatives regarding the extent to which they felt valued, respected and confident in their PPI role [59, 60]. Feelings of frustration and apprehension have been reported as barriers to resident involvement in PPI research activities, whereas feeling valued and trusting relationships were identified as facilitators [16]. While these findings may be relevant, it is unclear whether the same factors would apply to co-production activities, which seek to address power imbalances, and to the multiple stakeholder groups in care home settings. This is another area for future research.

Barriers and facilitators to co-production in care home research were identified. These may be helpful for planning future co-production approaches in this context. Our review adds to the existing literature by identifying barriers and facilitators that are specific to co-production in care home settings, such as consideration of care home resident characteristics and the potential burden on care home staff. Flexibility and early engagement of care home residents and staff will be needed to plan co-production activities in a way that is inclusive of their needs. Some of the factors identified align with key considerations identified from co-production research in wider health settings, including the importance of support from management and frontline staff, researchers’ skill sets (building relationships, managing expectations, negotiation, flexibility), and the impact of traditional academic practice and logistical arrangements on facilitating authentic co-production [9]. This suggests that greater investment in training and programme funding, and reviewing academic structures, such as governance processes and resource allocation, may be required if authentic co-production is the intended aim.

### Strengths and limitations

There is increasing interest in the use of co-production in social care settings and the review is timely to support researchers in undertaking co-production activities in care home contexts. A collaborator group of care home and health care representatives were involved in decision-making regarding the presentation and implications of findings from their perspectives. The review was completed in line with the JBI scoping review methodology [19] and PRISMA-ScR reporting guidelines [20] to aid transparency and rigour. No data restrictions were applied which allowed us to capture the breadth of available published literature. All studies were independently screened by two reviewers, and data charting and synthesis involved multiple reviewers to enhance replicability.

There are several limitations to the review. The search strategy may not have identified all studies due to the exclusion of non-English studies and grey literature. Our decision to include only papers that explicitly stated they had used co-production, co-creation or co-design may have excluded relevant studies using wider terminology. These terms were not used in social care research papers published before 2013 therefore the review may have missed earlier studies that used approaches in keeping with the principles of co-production. However, as these terms are already unclear concepts, we were mindful of not adding to this confusion and muddying the implications of the review’s findings. Although data charted was checked by a second reviewer, modification of the data charting table using a reporting checklist that was developed from a small number of co-creation case studies may have led us to overlook important components of co-production approaches that were not included in this. Our focus on published research may mean that the findings from the included studies are written from researchers’ perspectives for an academic audience therefore the review findings may not be representative of the views of the diverse stakeholders involved in co-production approaches in care homes. Another limitation is that the study collaborator group involved in this review does not include representation of current care home residents therefore the interpretation of the review findings may not be reflective of residents’ perspectives. Please see Additional file 4 for further information about our attempts to involve residents in this project and our reflections on this experience.

## Conclusions

This review has identified studies which used co-production approaches in a care home setting. A diverse range of approaches to co-production were used and a wide range of stakeholders participated at various stages and levels, including staff and residents. Barriers and facilitators to achieving authentic co-production in care home settings were also identified. The components, barriers and facilitators identified should be used to inform future co-production research. Future studies should be explicit in reporting what is meant by co-production, the methods used to support co-production and the extent to which co-production was achieved. Sharing of key learning is required to support this field to develop. Evaluation of co-production processes from diverse perspectives, including stakeholders’ experiences of co-production, are areas for future research in care home settings.

## Supplementary Information

**Additional file 1.** PRISMA-ScR checklist

**Additional file 2.** Example search strategy for MEDLINE

**Additional file 3.** Data charting table

**Additional file 4.** GRIPP2 reporting form

## Data Availability

The datasets used and/or analysed during the current study are available from the corresponding author on reasonable request.

## Abbreviations

CCG: Clinical Commissioning Group
JBI: Joanna Briggs Institute
PCC: Population, concept, context
PPI: Patient and Public Involvement
PRISMA-ScR: Preferred Reporting Items for Systematic reviews and Meta-Analyses extension for Scoping Reviews
