# Supplementary material for: Approaches to co-production of research in care homes: a scoping review
Source: Res Involv Engagem. 2022 Dec 23;8:74. doi: 10.1186/s40900-022-00408-z (PMC9780102; doi:10.1186/s40900-022-00408-z)
Supplement: Supplementary file 2 — Additional file 2. Example search strategy for MEDLINE [file 40900_2022_408_MOESM2_ESM.docx]

**Database:**
Ovid MEDLINE(R) ALL <1946 to December 17, 2021>

| **#** | **Query** | **Results from 20 Dec 2021** |
| --- | --- | --- |
| 1 | Community-Based Participatory Research/ | 5,166 |
| 2 | Community Participation/ | 18,011 |
| 3 | (co-produc* or co-creat* or co-design* or coproduc* or cocreat* or codesign*).mp. [mp=title, abstract, original title, name of substance word, subject heading word, floating sub-heading word, keyword heading word, organism supplementary concept word, protocol supplementary concept word, rare disease supplementary concept word, unique identifier, synonyms] | 9,080 |
| 4 | Residential Facilities/ | 5,679 |
| 5 | Homes for the Aged/ | 14,566 |
| 6 | Nursing Homes/ | 37,411 |
| 7 | ("care home" or "care facilit*" or "care institut*" or "long-term care" or "long term care").mp. [mp=title, abstract, original title, name of substance word, subject heading word, floating sub-heading word, keyword heading word, organism supplementary concept word, protocol supplementary concept word, rare disease supplementary concept word, unique identifier, synonyms] | 95,151 |
| 8 | Aged/ | 3,298,914 |
| 9 | ("older people" or "older person" or "older adult*" or "aged" or "elder*" or "senior*").mp. [mp=title, abstract, original title, name of substance word, subject heading word, floating sub-heading word, keyword heading word, organism supplementary concept word, protocol supplementary concept word, rare disease supplementary concept word, unique identifier, synonyms] | 5,852,373 |
| 10 | 1 or 2 or 3 | 31,660 |
| 11 | 4 or 5 or 6 or 7 | 131,653 |
| 12 | 8 or 9 | 5,852,373 |
| 13 | 10 and 11 and 12 | 223 |
