# Supplementary material for: Approaches to co-production of research in care homes: a scoping review
Source: Res Involv Engagem. 2022 Dec 23;8:74. doi: 10.1186/s40900-022-00408-z (PMC9780102; doi:10.1186/s40900-022-00408-z)
Supplement: Supplementary file 4 — Additional file 4. GRIPP2 reporting form [file 40900_2022_408_MOESM4_ESM.docx]

# Patient and Public Involvement in the Study using the GRIPP2 Short Form

| Section and topic | Item |
| --- | --- |
| 1: Aim  Report the aim of the study | To map co-production approaches used in care homes for older adults to inform the design of future co-production research |
| 2: Methods  Provide a clear description of the methods used for patient and public involvement (PPI) in the study | A study collaborator group contributed to the design, processes and documentation of the NIHR Advanced Fellowship programme in which this scoping review sits. Over the course of the Fellowship programme, the study collaborator group has consisted of a care home manager, a care home quality lead, an individual with past experience as a carer and care home resident, a care home matron and a consultant therapist in falls prevention. Findings from the review were shared and discussed with the study collaborator group at an online group meeting which was attended by the care home quality lead, the individual with past experience as a carer and resident, the care home matron and the consultant therapist. |
| 3: Results  Outcomes—Report the results of PPI in the study, including both positive and negative outcomes | The study collaborator group contributed to the presentation of findings and their implications as follows:  Design of the carousels of co-production diagram (figure 4)  Selection of accessible terminology to describe the process of co-production  Identification of areas for future research- the group highlighted the lack of findings relating to co-researcher/stakeholder experiences of how it feels to be working in the space of co-production |
| 4: Discussion  Outcomes—Comment on the extent to which PPI influenced the study overall. Describe positive and negative effects | The involvement of the collaborator group influenced important aspects of this review as outlined in section 3. The study collaborator group informed the interpretation of findings based on their unique perspectives and lived experiences of engaging in research and working collaboratively in care home settings. The study collaborator groups’ knowledge of stakeholders who may be potentially interested in the findings of this review, such as care home staff and residents, influenced the presentation of findings in a way that is accessible to key audiences outside of academia. However, despite our attempts to engage care home residents as part of the study collaborator group, no current care home residents were recruited to the group therefore their perspective is lacking from this review. |
| 5: Reflections  Critical perspective—Comment critically on the study, reflecting on the things that went well and those that did not, so others can learn from this experience | This study demonstrates the importance of seeking diverse perspectives in order to maximise learning from the review findings and to identify research gaps which are meaningful to those involved in care home settings. Conducting online group meetings worked well for this established group who are experienced in working together in this way, live in multiple locations across England and have limited time available to attend in-person meetings. However, this approach may not have been suitable for individuals with less experience of involvement in research advisory roles and online approaches presented challenges for involving current care home residents. We tried various routes to access care home resident input, contacting care homes directly and through colleagues with access to established care home networks. Two residents from one care home who were potentially interested in our research programme were identified via a care home manager; however, we were unable to visit the care home in person because of risks associated with the Covid-19 pandemic. We arranged for care home staff members and local care home research facilitators to support an online call with the residents using an IPad. However, despite multiple attempts, each time the call was rearranged due to unexpected demands on care home staff’s time and closures of the home. If we were to repeat the process, we would extend our timescales for seeking resident input. Resident involvement in co-production is an area we plan to explore further in future components of this research programme. |
